# An algorithm for separation of mixed sparse and Gaussian sources

**DOI:** 10.1371/journal.pone.0175775

**Published:** 2017-04-17

**Authors:** Ameya Akkalkotkar, Kevin Scott Brown

**Affiliations:** 1 Department of Chemical and Biomolecular Engineering, University of Connecticut, Storrs, CT, United States of America; 2 Department of Biomedical Engineering, University of Connecticut, Storrs, CT, United States of America; 3 Departments of Physics, and Marine Sciences, University of Connecticut, Storrs, CT, United States of America; 4 Institute for Systems Genomics and CT Institute for the Brain & Cognitive Sciences, Storrs, CT, United States of America; University of Rijeka, CROATIA

## Abstract

Independent component analysis (ICA) is a ubiquitous method for decomposing complex signal mixtures into a small set of statistically independent source signals. However, in cases in which the signal mixture consists of both nongaussian and Gaussian sources, the Gaussian sources will not be recoverable by ICA and will pollute estimates of the nongaussian sources. Therefore, it is desirable to have methods for mixed ICA/PCA which can separate mixtures of Gaussian and nongaussian sources. For mixtures of purely Gaussian sources, principal component analysis (PCA) can provide a basis for the Gaussian subspace. We introduce a new method for mixed ICA/PCA which we call **M**ixed **I**CA/**P**CA via **Re**producibility **St**ability (MIPReSt). Our method uses a repeated estimations technique to rank sources by reproducibility, combined with decomposition of multiple subsamplings of the original data matrix. These multiple decompositions allow us to assess component stability as the size of the data matrix changes, which can be used to determinine the dimension of the nongaussian subspace in a mixture. We demonstrate the utility of MIPReSt for signal mixtures consisting of simulated sources and real-word (speech) sources, as well as mixture of unknown composition.

## Introduction

Trying to infer underlying source signals present in a complex signal mixture is a ubiquitous problem in signal processing with applications across science and engineering. The classic example is the so-called “cocktail party problem,” in which the goal is to recover the voices of individuals speaking simultaneously using recordings from ambient microphones placed throughout the room [1]. In most cases, very little information about the underlying source signals is known; algorithms to attempt to solve this problem go under the heading of blind source separation [2]. Independent Component Analysis (ICA) is a blind source separation method that uses statistical independence of the sources as a criterion for solving the unmixing problem. The sources and mixing coefficients produced by ICA when multiplied together recover the data matrix. This is similar to the kind of matrix decomposition obtained via principal component analysis (PCA) [3], which is not surprising given that ICA reduces to PCA if the assumption of statistical independence of the sources is relaxed to the weaker condition of linear decorrelation. PCA is so widely used that is has been reinvented multiple times under different names (empirical orthogonal function analysis [4], the Karhunen-Loeve transform [5], and proper orthogonal decomposition [6]). ICA is used in many application domains [7, 8], particularly in neuroimaging, in which the goal is to decompose electroencephalographic (EEG) data in temporally independent sources [9] and functional magnetic resonance imaging (fMRI) data into spatially independent brain networks [10]. Two of the most common algorithms for ICA are maximum-likelihood [11] and minimization of the between-component mutal information [2]. Another frequently used neural network method (infomax [12, 13]) is equivalent to maximum-likelihood [14].

Unfortunately, once Gaussian sources are mixed with nongaussian sources ICA encounters problems. The unmixing matrix loses uniqueness because of the rotational invariance of the Gaussian subspace; with only nongaussian sources uniqueness is preserved [15]. Once two or more Gaussian sources are present in the signal mixture ICA can no longer separate those sources, and ignoring these sources in the ICA model will result in spurious sparse sources. This sphericity problem led Woods et al. [15] to propose a model for mixed ICA/PCA. They maximize an explicit likelihood model that incorporates supergaussian, subgaussian, and Gaussian sources and use cross-validation to determine the appropriate number of components of each kind. The method performs well but with a huge computational burden. Cross-validation alone is computationally expensive, and multiple likelihood maximizations are required for each model. A combinatorially large number of models must be evaluated, making this method difficult to use on the kinds of high-dimensional mixtures common in many application domains. Concerns about computational efficiency in ICA calculations have made FastICA [16], a fast fixed-point algorithm for ICA, an extremely popular method for source separation. Another of its strengths, relative to explicit likelihood maximization, is the fact that it can relatively easily separate mixtures of sources with both positive (subgaussian) and negative (supergaussian) kurtosis, without having to specify in advance how many of each are likely present.

RAICAR (Ranking and Averaging Independent Component Analysis by Reproducibility) [17] is an ICA method that uses repeated FastICA realizations to rank and select components based on their reproducibility, a measure of realization-to-realization consistency for a particular extracted source. It forms the basis for BICAR [18, 19], an ICA-based algorithm for multiresolution spatiotemporal data fusion. ICA decomposition of Gaussian mixtures produces purely spurious sparse components that do not stably converge as sample size increases. This property suggests that what reproducibility may be measuring is the degree to which a particular extracted component is part of a Gaussian subspace. This led Woods et al. [15] to speculate that a technique like RAICAR could be used to provide information for model selection in ICA. Unfortunately, as we will show, reproducibility alone is insufficient in determining how many Gaussian components are present in a complex signal mixture. However, component reproducibility along with a measure of reproducibility fluctuations across extractions from many random subsamples of the signal mixture matrix *can* identify the number of Gaussian and nongaussian sources in real mixtures.

In what follows, we describe a new algoritm for mixed ICA/PCA which we call MIPReSt: **M**ixed **I**CA/**P**CA via **Re**producibility **St**ability. Our method has RAICAR at its core, which allows it to take advantage of the speed and distributional flexibility of FastICA. While RAICAR itself requires multiple ICA runs, this number of decompositions does not grow with the size of the data matrix and FastICA is much faster than likelihood maximization. We demonstrate the performance of our algorithm on simulated mixtures of statistical sources, mixtures of real speech signals, and the famous Iris data of R.A. Fisher [20].

## Methods

### Algorithm

#### RAICAR

MIPReSt has at its core the RAICAR algorithm itself [17]; we use a modified version described previously [18, 19]. Briefly, in RAICAR the data matrix *X* is subjected to a *K*-fold FastICA [16] decomposition; each of the *K* FastICA realizations begins with random unmixing matrices (orthogonalized matrices of random Gaussian elements) and the same number of sources *N*_*s*_ are extracted in each. Matrices of source-source correlation coefficients are produced for each of the *K*(*K* − 1)/2 pairs of realizations, and components are grouped according to similarity across realizations. This re-sorts the estimated sources and mixing matrices from *K* sets of size *N*_*s*_ into *N*_*s*_ sets of size *K*. The average inter-group cross-correlation among the *K* sources in each of the *N*_*s*_ groups (either over all sources [18] or thresholded [17]) produces a value *R*, the reproducibility, which can be used to rank sources in descending order of statistical robustness.

#### MIPReSt

**Fig 1:**
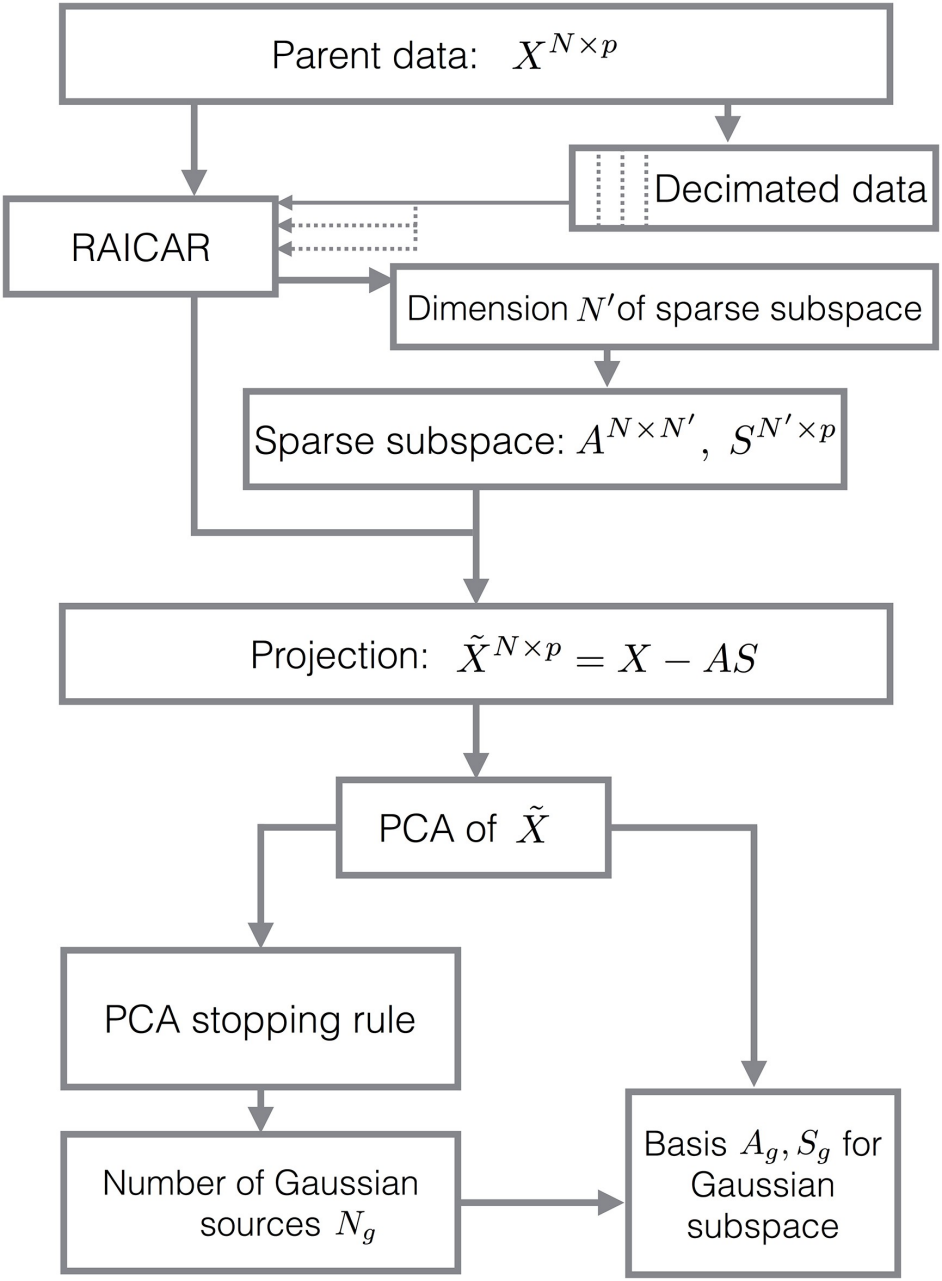
Schematic for MIPReSt. MIPReSt runs the RAICAR algorithm on both the original data matrix *X* and many random subsamples of smaller column dimension. Comparison of the reproducibilities from the original data and the random subsamples determines the size of the sparse subspace. After projecting that subspace out of *X*, singular value decomposition $\tilde{X}$, along with an eigenvalue selection rule, produces both the dimension of the Gaussian subspace and a basis for that subspace. (See Methods for details.).

In MIPReSt, the RAICAR algorithm becomes one step in a multi-step pipeline (see Fig 1). The key additional step is to perform RAICAR many times over many decimated versions of the original data. As discussed in the introduction, we expect the Gaussian subspace to be randomly oriented from subsample to subsample. We typically use multiple two-fold decimations only; results are similiar for multiple decimations of increased order (twofold, fourfold, eightfold, etc.) or multiple fourfold decimations alone. From each RAICAR run, we obtain ranked reproducibility values for all sources. We use these to compute another quantity *δ*_*ij*_ that measures decimation-to-decimation variability in the reproducibility, computed as

$$\delta_{ij}=\left|R\left(S_{j}^{i}\right)-R\left(S_{j}^{0}\right)\right|.$$ (1)

Here, *i* indexes realizations, with a superscript of 0 indicating reproducibility values obtained from a RAICAR decomposition applied to the parent (non-decimated) data matrix. *j* indexes sources, of which there will be *N* in all RAICAR runs.

Once we have used the decimation results to identify the dimension *N*′ of the sparse subspace, we project it out of the parent data matrix via

$$\tilde{X}=X-AS,$$ (2)

in which *A* (size *N* × *N*′) and *S* (size *N*′ × *p*) are the portions of the mixing matrix and sources arising from the RAICAR decomposition of the *parent* data matrix. The matrix $\tilde{X}$ now consists of only Gaussian components. The dimension of this residual Gaussian subspace will be no more than *N* − *N*′. A basis for the Gaussian subspace can be obtained via singular value decomposition of $\tilde{X}$, as described below.

#### Basis for the Gaussian subspace

Estimating the dimension of the Gaussian subspace is equivalent to deciding how many principal components to retain when performing PCA on the data matrix [3]. There are a large number of proposed PCA stopping rules, both heuristic and statistical, which have been reviewed and compared elsewhere [21, 22]. No clear consensus yet exists as to which (if any) rule is superior, likely because the ability of a particular rule to stop at the correct number of true components depends on the correlation structure in the data and size of the data matrix [22]. We therefore compare dimensionality estimates from six different rules, described below. Unless otherwise noted we begin with a singular value decomposition of the sample covariance matrix *C* = *YY*^*T*^/(*p* − 1), where *Y* is a row-centered version of $\tilde{X}$ and *p* is the column dimension of $\tilde{X}$. Eigenvalues *λ*_1_, …, *λ*_*n*_ are assumed ordered from largest to smallest.

Kaiser-Guttman (KG) Criterion The Kaiser-Guttman selection rule [23] is one of the simplest and most widely-used rules, despite its known shortcomings [24]. To use Kaiser-Gutman, calculate $\bar{\lambda }=\left(1/n\right)\sum_{i}\lambda_{i}$ and keep all components for which $\lambda_{i}>\bar{\lambda }$.
Joliffe-Modified KG Joliffe has proposed a modification of the KG criterion which accounts for sampling variance [3]. In Joliffe’s method, retain all components for which $\lambda_{i}>0.7\bar{\lambda }$.
Broken Stick Model The broken stick model began as a resource distribution model in ecology [25], and was only later applied to eigenvalue selection in PCA by associating the resource to apportion with the total variance in the data [26, 27]. Broken stick partitions the unit interval into *n* subintervals of random length, using *n* − 1 division points uniformly sampled in [0, 1]. If the subintervals are arranged in order of largest to smallest, then the expected value for the length of the *k*^th^ subinterval is
  $$l_{k}=\frac{1}{n}\sum_{i=k}^{n}\frac{1}{i}.$$ (3)
  To use the broken stick model for eigenvalue selection, first transform *C*’s eigenvalues to *f*_*k*_ = *λ*_*k*_ / ∑*λ*_*k*_ and then compare *f*_*k*_ to the values *l*_*k*_ from the broken stick distribution. Component *k* is retained if *f*_*k*_ is greater than *l*_*k*_.
Information Dimension Information dimension is a heuristic measure of the number of “informative” modes in PCA. Full details and motivation can be found elsewhere [21]. Briefly, it begins by converting eigenvalues to “probabilities” via *p*_*k*_ = *λ*_*k*_/∑_*k*_ *λ*_*k*_. These probabilities are then used to calculate a normalized entropy $\tilde{H}=-\sum_{k}p_{k}\log_{2}p_{k}/\log_{2}N$, where *N* is the row (or column) dimension of the covariance matrix. The information dimension *n*_0_ of the data is computed as $n_{0}=N^{\tilde{H}}$.
Parallel Analysis (PA) Horn’s parallel analysis criterion [28] compares the observed eigenvalues to the eigenvalues obtained from random matrices consisting of standard Gaussian random variables. First, standardize each variable in $\tilde{X}$ so *C* becomes the correlation (and not covariance) matrix. Then generate 10^3^ matrices of the same dimensions as $\tilde{X}$ with *N*(0, 1) entries. Obtain critical values using a predetermined significance level, and stop retaining components once the real data eigenvalues drop below the critical values. We use a 95% significance level to calculate critical values.
Random Lambda This method is a permutation test for each eigenvalue/component [29]. The values within $\tilde{X}$ are randomly shuffled 999 times and eigenvalues are computed each time. A permutation *p*-value is computed via *p* = (*n* + 1)/1000, where *n* is equal to the number of times a random eigenvalue was larger than its corresponding data value. We then discard any components for which *p* > 0.05.

Once the dimension of the Gaussian subspace has been computed, a basis (set of sources) for the Gaussian subspace can be obtained by projection of the data matrix onto the subspace spanned by the retained eigenvectors. A python package for MIPReSt will be available on Github (https://github.com/thelahunginjeet); it depends on other packages which are also all available at the same location.

### Data

We used three types of data: simulated sources, speech signals extracted from public-domain audiobooks, and the famous Fisher’s Iris data [20]. Gaussian sources were always sampled from standard unit normal distributions, specifically

$$p\left(x\right)=\sqrt{\frac{1}{2\pi }}e^{-x^{2}/2}.$$ (4)

#### Simulated sources

We used several different distributions to generate subgaussian and supergaussian sources (see Table 1); some of these distributions have been used as test data previously [15]. All sparse sources were either generated from distributions with zero mean and unit variance or were standardized after construction.

**Table 1:** Simulated sparse sources used in this study.

Name	Distribution	Type
Inverse Cosh	${\left(2\text{ cosh}\left(\frac{\pi x}{2}\right)\right)}^{-1}$	super
Laplace	$\frac{1}{\sqrt{2}}e^{-\sqrt{2}\left|x\right|}$	super
Logistic	$\frac{\pi }{4\sqrt{3}}{\mathrm{sech}}^{2}(\frac{\pi x}{2\sqrt{3}})$	super
Exponential ArcSinh	$\frac{1}{\sqrt{2\pi \alpha^{2}{\left(1+x/\alpha \right)}^{2}}}\exp(-{\mathrm{arcsinh}}^{2}(\frac{x}{\alpha })/2)$	super
Double Cosh	$\frac{1}{\sqrt{e\pi }}e^{-x^{2}/2}\cosh(x\sqrt{2})$	sub
Exponential Sinh	$\sqrt{\frac{1+\sinh^{2}\left(x\right)}{2\pi }}\exp(-\frac{\sinh^{2}\left(x\right)}{2})$	sub
Generalized Gaussian	$\frac{\beta }{2\Gamma (1/\beta )}e^{-{\left|x\right|}^{\beta }}$	super for 0 < *β* < 2, sub for *β* > 2

#### Speech

**Fig 2:**
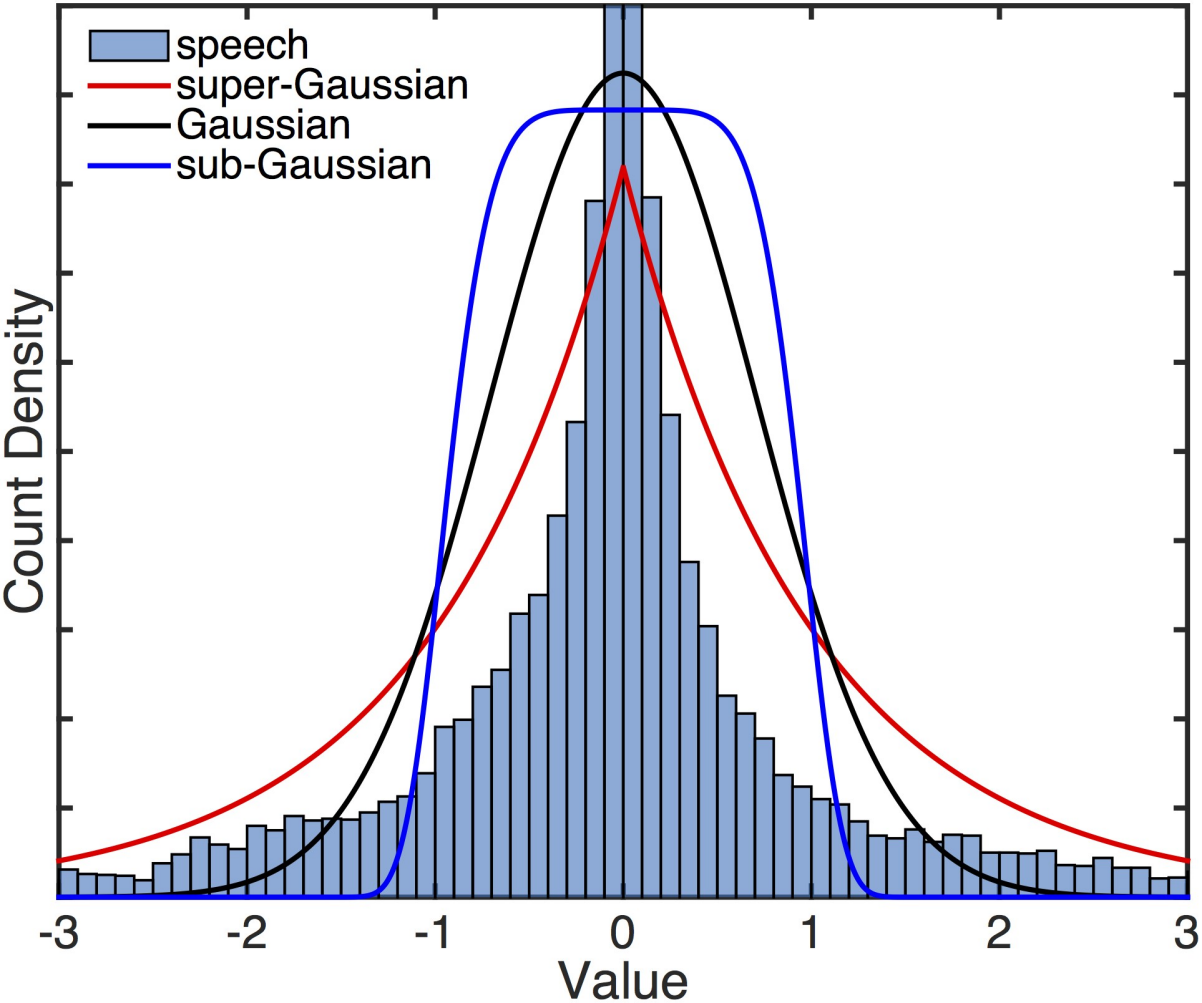
Examples of super- and subgaussian sources. Shown here are histograms for a Gaussian source (black), a subgaussian source (the generalized Gaussian), and a supergaussian source (Laplace). Also shown is a histogram for one of the speech signals used in this study. The speech signal is far more leptokurtic than the Laplace source; without truncating the *y*-axis the massive spike near zero of the speech signal obscures the shapes of the other distributions.

We obtained five mp3s of public domain audiobooks from librivox.com [18]. The works used were “History of the Peloponnesian War”, Book 5 by Thucydides; “Flatland” by Edwin A. Abbott; “The Adventures of Huckleberry Finn” by Mark Twain; The “Confessions” of St. Augustine; and “Moby Dick” by Herman Melville. These audiobooks were converted from stereo to single channel (mono) when appropriate, and then downsampled to 2.75 kHz. Examples of supergaussian and subgaussian distributions and a histogram of a random five second audiobook segment are shown in Fig 2.

#### Iris data

R.A. Fisher’s famous Iris dataset [20] is available for download at the UCI Machine Learning Repository [30]. With the exception of ignoring the class labels in the data file no additional processing (beyond standard ICA preprocessing) of this data was peformed.

## Results

### Full rank extraction

As we discussed in the introduction, the Gaussian subspace in a mixture of sparse and Gaussian signals should randomly orient as the number of samples increases. To motivate the MIPReSt algorithm, we performed the following numerical experiment. We generated a five-dimensional signal mixture consisting of one supergaussian source (Inverse Cosh), one subgaussian source (Double Cosh), and three Gaussian sources (see Table 1 for these super- and subgaussian distributions). Each source consisted of 5 × 10^5^ standardized iid samples, and we mixed them using a random 5 × 5 orthogonal matrix. We then randomly subsampled this parent signal mixture to obtain signal mixtues consisting of between 5 × 10^3^ and 5 × 10^5^ samples. We applied the RAICAR algorithm to each of the signal mixtures, and we calculated reproducibilities for each RAICAR source in every mixture. In order to match each of the five RAICAR sources to a unique known input source to which it was most similar, we solved the linear assignment problem [31] using Munkres’ version of the Hungarian algorithm [32]. The cross correlations between the RAICAR sources and the known sources were used as the basis of the cost matrix for the assignment problem.

**Fig 3:**
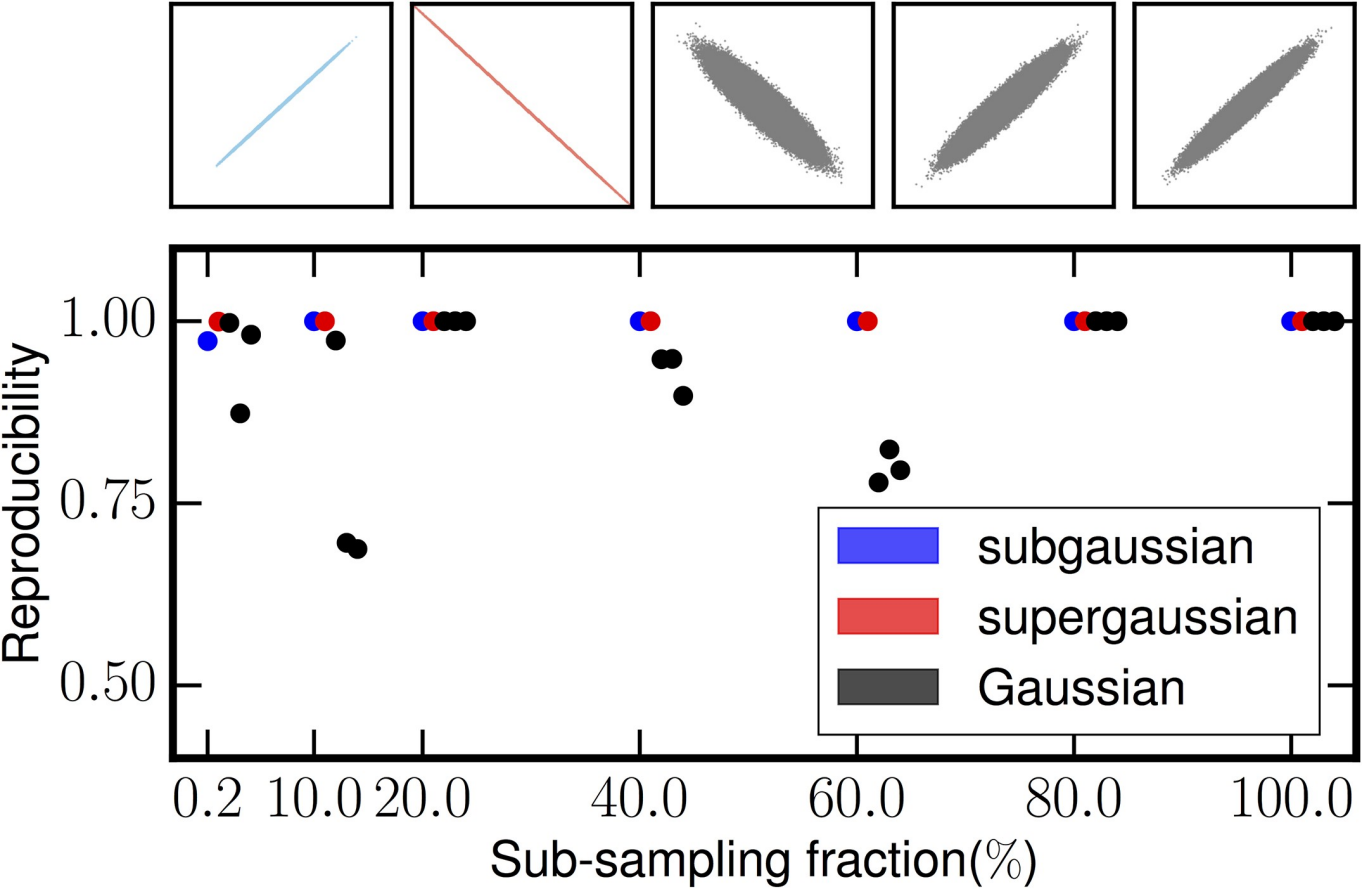
Full rank extraction. We constructed a simulated data matrix with five sources: one supergaussian, one subgaussian, and three Gaussian sources. The simulated data matrix had 5 × 10^5^ samples. The main panel shows the results of RAICAR extractions at different levels of decimation, including the parent data. The best assignment match to the supergaussian source is shown in blue and to the subgaussian source in red. While the Gaussian sources may sometimes have extrememly high reproducibility, they show poor stability when the data is decimated, in constrast to the sparse sources. The top panel shows scatter plots of the estimated sources from the parent data against their best assignment match; the sparse sources are recovered perfectly by RAICAR.

Fig 3 shows the results of these calculations. In each set of five reproducibilities, one set per decimated data set, the blue point corresponds to the best assignment match to the known supergaussian source, the red point corresponds to the best match to the subgaussian source, and all the Gaussian sources are shown in black. The inset shows the RAICAR sources from the parent data set of 5 × 10^5^ samples plotted against their best assigment match. There are several things of note in this figure. First, RAICAR finds that the nongaussian subspace is highly, and usually perfectly, reproducible even at far more modest sample sizes than in the parent data. Secondly, the Gaussian subspace does orient randomly, as shown by the fluctuating Gaussian reproduciblities. Third, the Gaussians sometimes have extremely high reproducibility, which indicates that *reproducibility alone cannot discriminate the Gaussian subspace from the nongaussian subspace*. These three observations motivate the core of the MIPReSt algorithm. We perform RAICAR on many subsampled versions of the input data. We monitor not only the distribution of reproducibilities *R*, but also the decimation-to-decimation variations in reproducibility *δ*_*ij*_ defined in Eq 1. True sparse sources will tend to have uniformly high *R* and low *δ*_*ij*_, while Gaussian sources may sometimes have high *R* but also larger *δ*_*ij*_.

### Overextraction

In most cases, the total signal dimension—the total number of true sources of any kind—is unknown and must be either estimated from the data matrix or guessed. We have previously found that repeated estimation methods like RAICAR and BICAR [18, 19] are relatively robust to overestimation of the data dimension. For example, if the signal mixture is seven dimensional but the number of true sources is five, extracting seven sources yields two sources (which we will call spurious) with extremely low reproducibility. This suggests a simple protocol when confronted with a real signal mixture: extract as many sources as possible, up to the row dimension of the data matrix, and then use source reproducibility to estimate the total source content.

We performed a test to determine how MIPReSt performs for overextraction. Specifically, we wanted to know if spurious sources were clearly distinguishable in *R*, *δ*_*ij*_ space from both Gaussian and nongaussian sources. We therefore generated five input sources; two supergaussians (both Inverse Cosh) and three Gaussian sources, each of which had 5 × 10^5^ samples. (Using two subgaussians or one subgaussian and one supergaussian yielded identical results.) These were then mixed with a 10 × 5 Gram-Schmidt orthogonalized mixing matrix to obtain a ten dimensional data matrix which consists of only five real sources, Gaussian or otherwise. We applied MIPReSt to this mixture; in each case we used an ensemble of fifteen random 2-fold subsampled data matrices.

**Fig 4:**
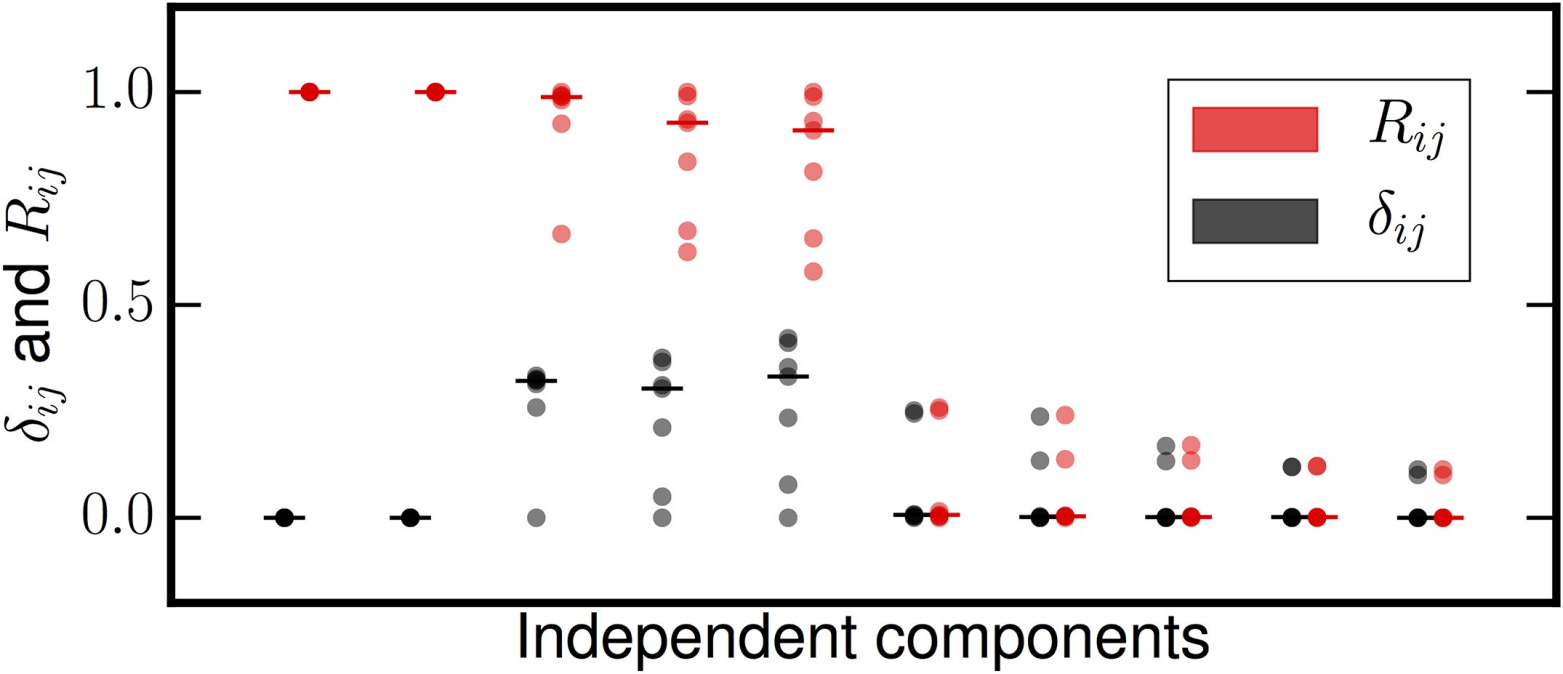
Reproducibility (*R*) and reproducibility fluctuations (*δ*_*ij*_) from overextraction. Only five sources (Gaussian or otherwise) are present, but the mixture dimension is ten. Horizontal bars are located at the median value. There are clearly three groups of sources here. Two sources (the recovered sparse sources) have near-perfect *R* that does not fluctuate from decimation-to-decimation. Three sources have occasionally high reproducibility, but also significant *δ*_*ij*_; these are the Gaussian subspace. The remaining five sources have very low reproducibility that fluctuates very little; these sources are spurious sources resulting from overextraction.

Fig 4 shows the results of this experiment. Based on the *R* and *δ*_*ij*_ values the extracted sources sort themselves into three categories: (*i*) signals with near unit reproducibility and zero delta, (*ii*) signals with high reproducibility but also high subsample-to-subsample fluctuations, and (*iii*) signals with very low reproducibiliy that does not fluctuate very much from subsample to subsample. Comparison of these three sets of sources to the known input sources by solving the assignment problem shows that the nongaussian sources are contained in the first group, all the Gaussian sources are in the second, and all spurious sources are completely unreproducible. In some subsamples, FastICA exhausts the variance in this data with fewer than five spurious sources; these missing spurious sources are assigned a reproducibility of zero. When we project out the recovered sparse sources and estimate the dimension of the Gaussian subspace, all PCA stopping rules arrive at the correct dimension of three (see Table 2). Based on this analysis, MIPReSt can recover the true sparse sources and the correct basis dimension for the Gaussian subspace even if the extraction dimension is larger than the true data dimension.

**Table 2:** Results for estimated dimension of Gaussian subspaces.

N (matrix/Gaussian sources)	Overextraction	Speech	Fisher
	10/3	15/5	4/?
KG	3	5	3
Joliffe KG	3	5	3
Broken Stick	3	5	3
Inf. Dim.	2.93	4.98	2.01
PA	3	5	2
Random Lambda	3	5	1

### Separation of complex, real-world signals

Next, we wanted to see if MIPReSt was still successful in recovering the dimension of the sparse subspace when that subspace consisted of sources with more realistic structure. We therefore continued to use simulated mixtures, but the nongaussian subspace was constructed from random sections of the public domain audiobooks described in Methods. Each nongaussian source consisted of 2 × 10^4^ contiguous audio samples, starting at a random location. At 2.75 kHz (see Methods) this consists of 7.3 seconds of audio. Each speech source was standardized. The simulated data matrices we constructed consisted of five such speech sources and five Gaussian sources. These were overmixed using a Gram-Schmidt orthogonalized random mixing matrix to a fifteen dimensional data matrix. We used fifty two-fold subsampled data matrices for MIPReSt calculations of *R* and *δ*_*ij*_.

**Fig 5:**
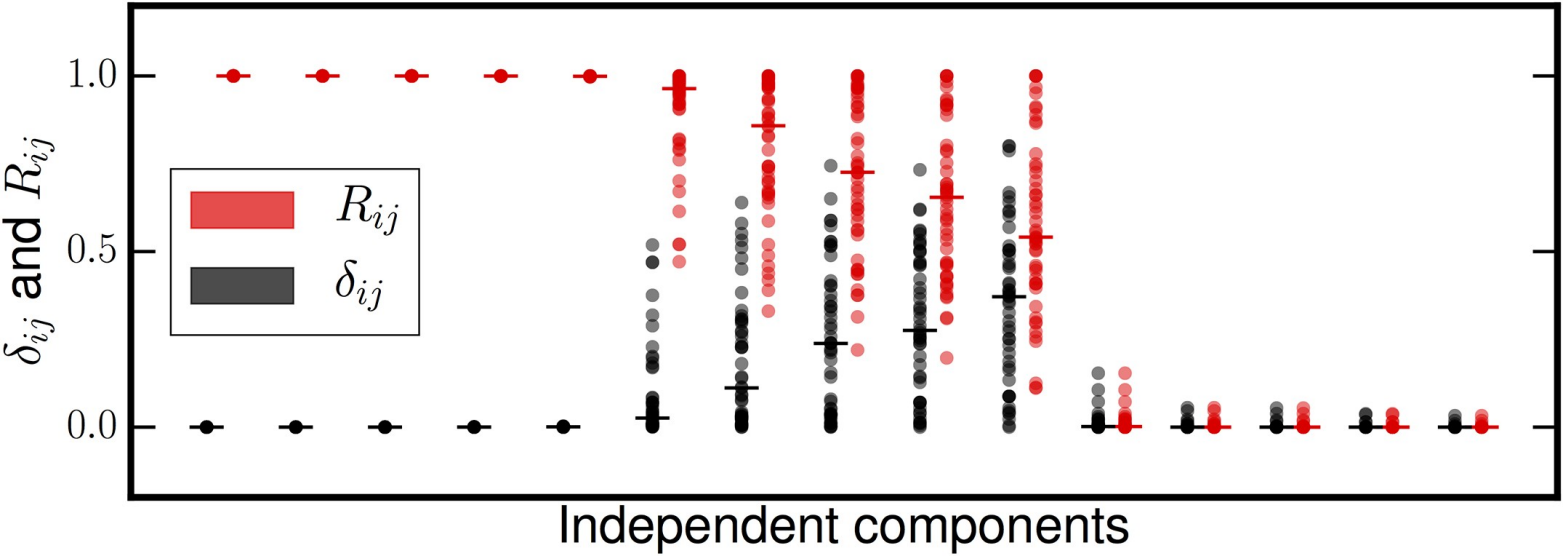
Reproducibility (*R*) and reproducibility fluctuations (*δ*_*ij*_) for speech signals mixed with Gaussian sources. For each of the fifteen extracted sources, *R* is shown in red and *δ*_*ij*_ in black. For both quantities, values for each of the fifty subsampled data matrices are shown as points and the median value as a horizontal bar. The sources clearly group into three categories: high *R* with low *δ*_*ij*_ (true sparse sources), variable *R* with high *δ*_*ij*_ (Gaussian sources), and low *R* and *δ*_*ij*_ (spurious sources).

Fig 5 shows the results of running MIPReSt on the overmixed speech examples. Again, as in Fig 4, one can see three distinct categories of sources, corresponding to the speech signals (high *R*, low *δ*_*ij*_), the five-dimensional Gaussian subspace (variable *R* but high *δ*_*ij*_), and the five spurious sources resulting from overmixing (low or zero *R* and low *δ*_*ij*_). This is the same pattern we saw in our previous experiments using simulated sparse sources. The sparse sources are near-perfectly reproducible and highly stable. The Gaussian components are occasionally very reproducible, but as before quite unstable to decimation. Finally any spurious sources related to overextraction have almost no reproducibility whatsoever. We performed multiple iterations of this experiment and the results were consistent every time. Contaminating each speech signal with Gaussian noise of varying signal-to-noise ratio had no effect on estimation of any of the subspace dimensions. This is expected; the added noise had identical characteristics to the signals making up the Gaussian subspace and hence caused no difficulties in extraction. As before, all PCA stopping rules agreed that the Gaussian subspace had a dimension of five.

### Fisher’s Iris data

Finally, we examined the performance of MIPReSt on Fisher’s famous Iris data set [20], originally introduced in the manuscript in which Fisher developed the linear discriminant. Fisher’s Iris data is probably the most famous classification dataset in existence; a partial list of papers which cite the Iris data, maintained on the UCI Machine learning Repository [30], contains over 200 papers. The Iris data has 150 samples measured on four dimensions (features): petal length, petal width, sepal length, and sepal width. In order to use MIPReSt on the Iris data, we subjected it to eighty random two-fold decimations. We emphasize that this data could be quite challenging for our method, as it has far fewer samples (by two or more orders of magnitude) than the test data we have considered previously.

**Fig 6:**
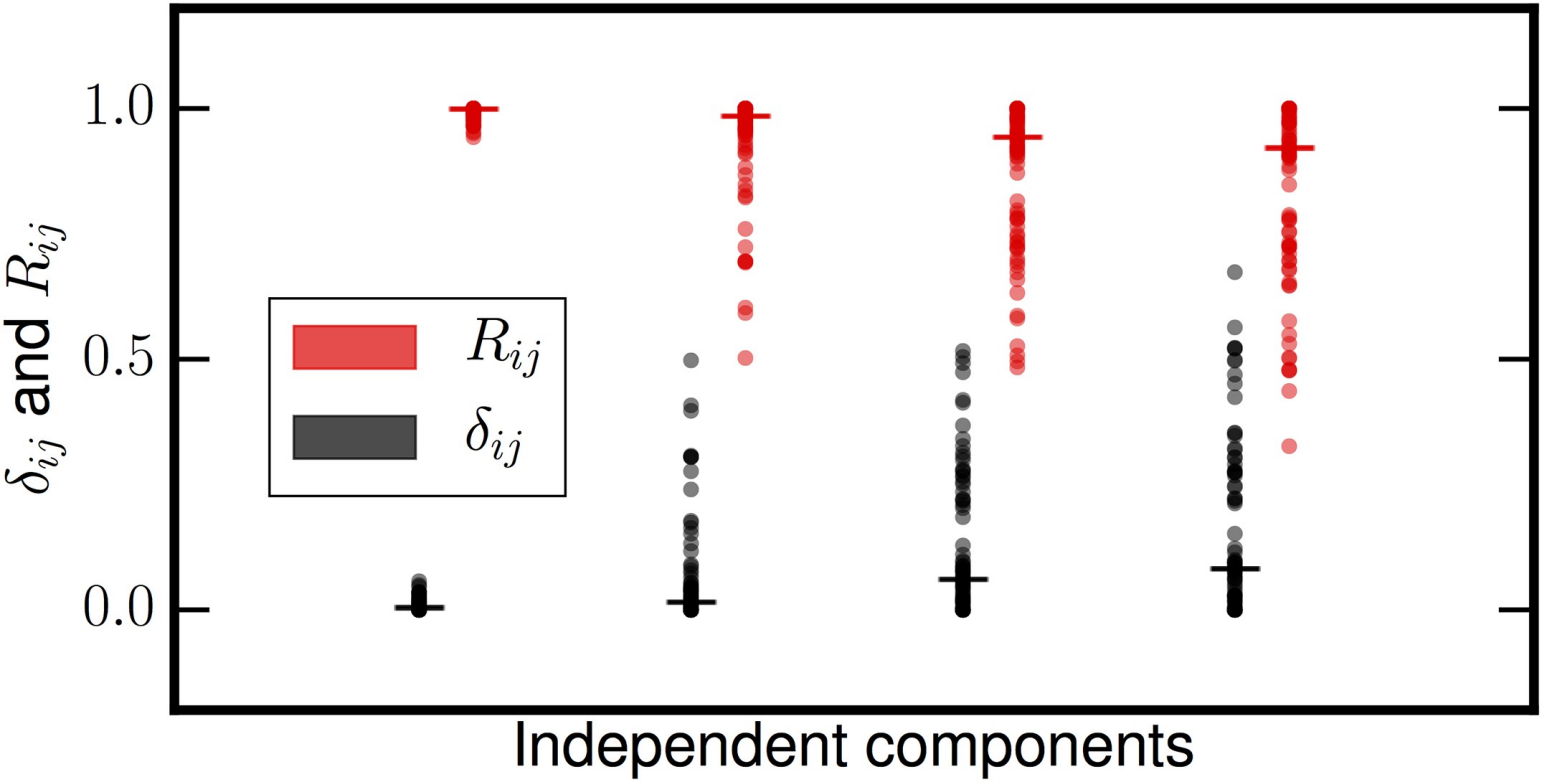
Reproducibility plot for the Iris data. The format and color scheme for this figure is identical to that used in Figs 4 and 5. Based on this information and related discussion in the text, it appears that there is one (and likely only one) sparse source present in the iris data.

**Fig 7:**
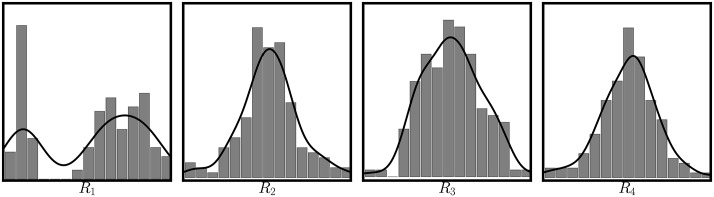
Histograms of extracted sources from the Iris data. Each panel shows a histogram (bars) and kernel density estimate (Gaussian kernel, solid line) for one of the four RAICAR sources extracted from the iris data. The nongaussianity of the most reproducible source (upper left) is clearly evident.

Fig 6 shows that there is one and only one sparse component present in the data. When we examine the RAICAR sources from the parent data in Fig 7 the nongaussianity of the high *R*_*ij*_, low *δ*_*ij*_ source is obvious (far left panel in the figure). We note that our estimate of a single sparse source in the Iris data agrees with that obtained by the algorithm of Woods et al. [15]. The fourth coulumn of Table 2 shows the estimates for dimension of the residual (after projection) Gaussian subspace. Here, there is less of a consensus than in the simulated mixture cases. The stopping rules indicate there are anywhere between 1 and 3 Gaussian sources present. Our reasons for including the Kaiser-Guttman criterion (and its modification by Joliffe) are its simplicity, speed, and wide use by practitioners. It has, however, been roundly criticized [24]. In a detailed simulation study, Peres-Neto and colleagues give high marks to PA and Random Lambda (along with four other rules we did not consider) [22]; these methods tend to produce the correct number of relevant components for the widest variety of correlation structures in the data. In addition, in a study of several stopping rules applied to microarray data [21] find similar disagreement and recommend a consensus approach based on multiple rules. If we exclude KG and Joliffe’s KG and simply average the results from the remainder of the rules, we obtain a Gaussian subspace dimension of two.

We therefore find that the Iris data, despite having a potentially problematic number of samples, did not pose a significant challenge to MIPReSt. We are able to unambiguously find only a single sparse source, a result that agrees with a previous mixed ICA/PCA method that requires a full likelihood model for the data [15]. These results, along with our decompositions of the audiobook speech signals above, strongly indicate MIPReSt will be a valuable algorithm for a variety of real-world data.

## Discussion

We have presented MIPReSt, a new algorithm for mixed ICA/PCA and demonstrated its utility for both simulated mixtures and empirical data. MIPReSt performs many repeated ICA realizations on both the original, parent data matrix *X* as well as a number of derived data matrices obtained from *X* via randomly dropping some fraction of the samples in *X*. Using a combination of component reproducibility and a measure of subsample-to-subsample fluctuations in reproducibility, we are clearly able to separate a complex mixture into sparse and Gaussian subspaces, as well as flag potentially spurious sources resulting from extraction of more sources than are actually present in the data. Even on data matrices with an extremely limited number of samples (150 for the Iris data), MIPReSt still obtains results consistent with other algorithms which are more heavily parameterized and much more computationally expensive [15]. In addition, MIPReSt’s use of FastICA allows it to recover both supergaussian and subgaussian sources without any need to specify the relative numbers of each.

As currently stands, MIPReSt uses a very basic version of FastICA. A single nonlinearity (logcosh) was used for all the data matrices we decomposed, and for every extracted source within those data matrices. Given that the sparse subspaces of real mixtures may be composed of sources with a variety of shapes, the use of a single nonlinearity may be questionable. Precisely these concerns have led others to develop a “reloaded” deflationary FastICA method that tries to adaptively find the optimal nonlinearity for each extracted source as the algorithm progresses [33]. This method showed improved performance over traditional FastICA when sources with varying distributional shapes were mixed together. It would be interesting and valuable to compare the performance of MIPReSt with this adaptive FastICA method to what we have used here. However, we should note that while gains could be made, they are not likely to be dramatic, given the performance of MIPReSt in this study. Using multiple estimations, we were able to recover non-Gaussian subspaces to high accuracy even when they consisted of a mixture of both super-Gaussian and sub-Gaussian components.

When dealing with datasets of much larger dimension, say those typical in ICA analyses of electroencephalographic [9] or functional magnetic resonance imaging [10] data, some sparse components may have reproducibility further from unity than we see here. This could cause a potential problem, since then the RAICAR averaged mixing matrix columns corresponding to these sources deviate from orthogonality. In these cases, it should be possible to either correct the mixing matrix to orthogonality via Gram Schmidt, or more simply use a single ICA realization on the parent data *X* in which we identify the true sparse sources and corresponding mixing matrix columns solving the assignment problem between the RAICAR/MIPReSt sources and the single-run ICA sources. As above, the cross-correlation matrix between the two sets of sources gives the cost matrix for the assignment problem.

Our method for estimation of the dimension of the Gaussian subspace relies on using one or more PCA stopping rules, an area in which there is guidance but not very much consensus [21, 22, 24]. We find very consistent results for dimension estimation in simulated mixtures, even when those mixtures contain real-world sources (speech). On Fisher’s Iris data, the results are less clear. The best approach would be to obtain a dimension estimate from a combination of stopping rules [21] that have proven to work well under a variety of correlation structures in the data [22]. However, we should point out here that for the applications considered here, obtaining the exact dimension for the Gaussian subspace is not really a concern. All we can recover is a basis for the Gaussian subspace, not the individal sources which compose it (which is impossible). In this case, it may actually be desirable to underestimate the dimension of the Gaussian subspace in order to obtain some amount of data compression.

In other cases, more careful evaluation of eigenvalue selection criteria will be warranted. If the Gaussian subspace were to consist of signals with non-identical power spectra—for example AR/ARMA models with nonidentical coefficients—then by using algorithms like SOBI [34] or AMUSE [35] we should be able to recover the constituent Gaussian processes and not just a basis for the subspace. In this case, estimation of the dimension of the residual data matrix $\tilde{X}$ becomes much more important, and a comprehensive study of the performance of eigenvalue selection algorithms for simulated mixtures of sparse and Gaussian sources will be necessary. We are currently working on a version of MIPReSt tailored to unmixing of time series and investigating this issue.

It has recently become much easier to collect EEG data from within an MRI scanner [36, 37], leading to the possibility of combining EEG and fMRI data during a cognitive task to obtain a single view of human brain activity with simultaneously high spatial and temporal resolution. Many methods have been proposed for this problem [38–46], and use of ICA as some part of the analysis or processing pipeline is a feature of many of these methods [18, 19, 36, 47–51]. However, none of these methods deal with the problem of nongaussian components in the data and the possible contamination of sparse sources during the ICA steps. We are currently working to integrate MIPReSt into BICAR, an ICA-based method for producing reproducible joint components from concurrent EEG-fMRI data [18, 19]. This should produce fewer spurious joint maps, and enhance the interpretability of the real ones.
